# A80 TIME-RESTRICTED FEEDING IMPROVES INFLAMMATORY AND METABOLIC BIOMARKERS IN CROHN’S DISEASE WITH OVERWEIGHT: A RANDOMIZED, PLACEBO-CONTROLLED PILOT TRIAL

**DOI:** 10.1093/jcag/gwae059.080

**Published:** 2025-02-10

**Authors:** N Haskey, A G Lewis, C Lavallee, M Yousuf, L Taylor, S Jilani, S Gold, C Lu, S Ghosh, R Panaccione, M Raman

**Affiliations:** Biology, The University of British Columbia, Vancouver, BC, Canada; Biology, The University of British Columbia, Vancouver, BC, Canada; University of Calgary, Calgary, AB, Canada; University of Calgary, Calgary, AB, Canada; LyfeMD, Calgary, AB, Canada; Biology, The University of British Columbia, Vancouver, BC, Canada; Icahn School of Medicine at Mount Sinai, New York, NY; University of Calgary, Calgary, AB, Canada; University College Cork, Cork, Cork, Ireland; University of Calgary, Calgary, AB, Canada; University of Calgary, Calgary, AB, Canada

## Abstract

**Background:**

Time-restricted feeding (TRF), a form of intermittent fasting (IF), has shown promise for weight loss and metabolic health. Up to 40% of patients with Crohn’s disease (CD) have a BMI >25, which together with increased visceral adipose tissue (VAT) are independent risk factors for increased hospitalizations, early surgical interventions, and reduced responsiveness to pharmacotherapy. Effects of IF regarding metabolic and inflammatory biomarkers in CD remain limited.

**Aims:**

To assess whether a TRF, compared to standard management (SM), affects inflammatory and metabolic biomarkers in CD patients with a BMI >25.

**Methods:**

We conducted a 12-week (12W) pilot randomized controlled trial, recruiting patients with CD and overweight from Calgary/Kelowna. Participants were randomly assigned in a 1:1 ratio to either TRF or SM. The TRF group fasted for 16 hours daily and ate within an 8-hour window (16:8) for 6 days per week, without altering their habitual diet. The SM group continued their habitual diet.

**Results:**

35 patients completed the protocol (TRF=20; SM=15). Baseline characteristics were similar between groups, with a median BMI 28.0 kg/m^2^ [IQR:26-33.1]; median age 49 years [IQR:37-60]; 63% female with a median fecal calprotectin (FCP) 115 mcg/g [IQR:42-543]. There were no significant differences between groups in terms of diet, clinical disease activity or inflammatory and metabolic biomarkers at baseline. At 12W, the TRF group experienced a significant reduction in BMI (-0.9 kg/m^2^, P=0.007) and VAT (-194 cm^3^, P=0.008) while the SM group saw an increase in BMI (+0.2 kg/m^2^) and VAT (+78 cm^3^). FCP levels were either maintained or reduced (P=0.046), serum amyloid A significantly decreased (P<0.0001), with a trend toward improvement in the Harvey Bradshaw Index (P=0.061) in the TRF group, whereas in the SM group FCP, serum amyloid A, and the Harvey Bradshaw Index increased over the 12W. Serum markers leptin (P=0.002), adipsin (P=0.023), and plasminogen activator inhibitor-1 (P=0.045), significantly decreased in the TRF group. These changes were not observed in the SM group; the SM group saw an increase in IL-6 levels over the 12 weeks (P=0.001).

**Conclusions:**

TRF led to significant improvements in BMI, VAT, and key metabolic markers, as well as decreases in systemic and gut inflammation, not observed in the SM group. These results highlight the potential of TRF as a complementary strategy for improving metabolic health and reducing inflammation in CD patients, warranting further investigation in larger studies. (NCT05230160).

**Funding Agencies:**

Crohn’s & Colitis Foundation

